# Point-of-Care Ultrasound for the Diagnosis of Colon Cancer

**DOI:** 10.24908/pocus.v7i2.15657

**Published:** 2022-11-21

**Authors:** Weihao Chen, Readon Teh, Absar Qurishi

**Affiliations:** 1 Division of Gastroenterology, Department of Medicine, Ng Teng Fong General Hospital Singapore; 2 Division of Gastroenterology and Hepatology, National University Hospital Singapore

**Keywords:** Pseudokidney, colorectal, cancer

## Abstract

We present a case of a 64-year-old gentleman for whom point of care ultrasound (POCUS) expedited the diagnosis and subsequent early treatment of colon adenocarcinoma. He was referred by his primary provider to our clinic for abdominal bloating. He had no other abdominal symptoms such as abdominal pain, change in bowel habits or rectal bleeding. He had no constitutional symptoms such as weight loss. The patient’s abdominal examination was also unremarkable. However, POCUS identified a 6 cm long hypoechoic circumscribed colon wall thickening around the hyperechoic pattern of bowel lumen (Pseudokidney sign)^1^ in the right upper quadrant, which suggested the presence of an ascending colon carcinoma. In view of this prompt bedside diagnosis, we organised a colonoscopy, staging computerised tomographic scan and colorectal surgery consultation the next day. After the locally advanced colorectal carcinoma was confirmed, the patient had curative surgery within 3 weeks of his presentation to the clinic.

## Case File

A 64-year-old gentleman with no significant past medical history except for hypertension was referred by his primary provider to our gastroenterology clinic for abdominal bloating. He had no other abdominal symptoms such as abdominal pain, change in bowel habits or bleeding per rectum. He had no constitutional symptoms such as weight loss. There was no family history of gastrointestinal malignancy but the patient was a chronic smoker. The patient’s physical examination was also unremarkable. A complete blood count done within the last 3 months was normal. 

**Figure 1:**
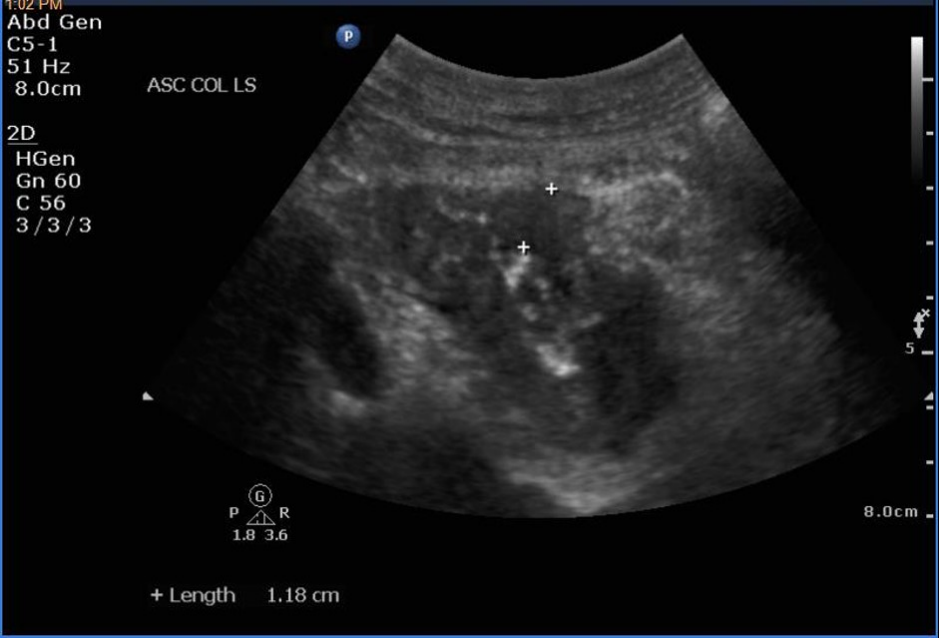
Ascending colon carcinoma. Probe place at right hypochondrium, probe marker pointing to patient’s head. Hypoechoic irregular circumscribed wall thickening (11.8mm) around the hyperechoic pattern of bowel lumen presenting with a kidney-shaped appearance by sonography. There is also loss of echo-stratification and compressibility.

Point-of-care ultrasound (POCUS), which is routinely performed at our clinics as an extension of our physical examination 2, detected a 6 cm long segment of hypoechoic irregular circumscribed colon wall thickening around the hyperechoic pattern of bowel lumen (Pseudokidney sign)^1^ in the right upper quadrant, which suggested the presence of a colon carcinoma in the ascending colon (Figure 1). 

**Figure 2:**
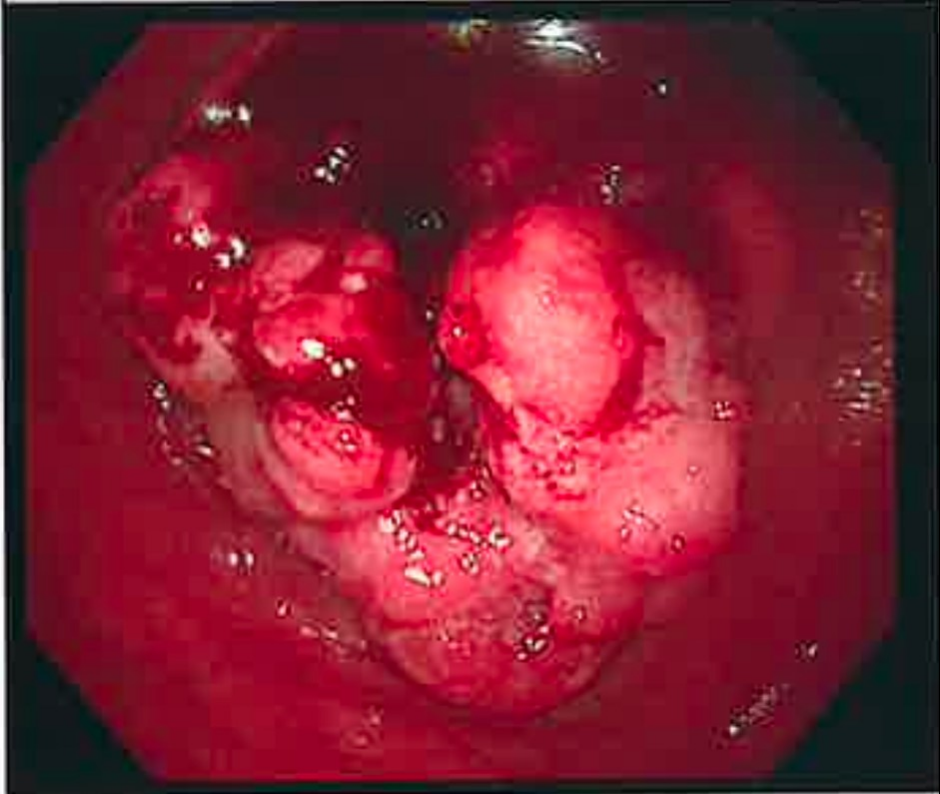
Colonoscopic picture: Circumferential exophytic tumour that caused luminal narrowing and was friable with contact bleeding. The biopsies were positive for the ascending colon adenocarcinoma.

**Figure 3:**
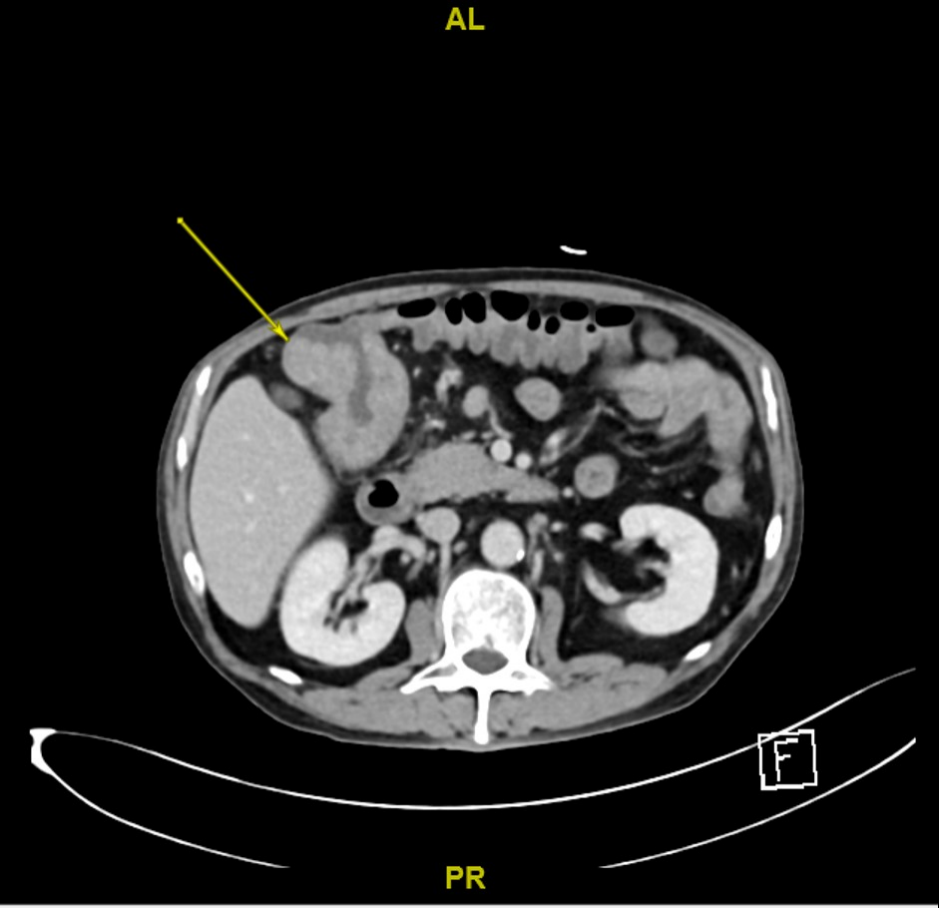
Axial CT scan showing a thickened loop of colon in the right hypochondrium.

In view of the POCUS findings, a decision was made to expedite the patient’s subsequent investigation and management. The patient went for a colonoscopy, staging computerised tomographic scan and colorectal surgery review the next day. Colonoscopy confirmed the presence of a circumferential exophytic tumour that caused luminal narrowing and was friable with contact bleeding (Figure 2). The histopathological findings were consistent with colon adenocarcinoma. Computed tomography (CT) was performed to determine the progression of the neoplastic process and the scan indicated ascending colon cancer without distal metastasis (T3N1M0) (Figure 3). After surgical consultation, the patient had curative surgery within 3 weeks of presentation.

## Discussion

Colorectal cancer (CRC) is the most common cancer in Singapore with an age standardised incidence rate of 39.1 per 100,000 population and age standardised mortality rate of 13.5 per 100,000 population. It is estimated that approximately 1287 new cases of colon cancer are diagnosed annually in Singapore 3. Prompt and accurate diagnosis of CRC is crucial to improve the prognosis of patients with CRC. Colonoscopy with biopsy is the gold standard of diagnostic methods for CRC. Unfortunately, during the COVID-19 pandemic, Singapore’s national CRC screening program was suspended from March to August 2020, which lead to a reduction of colonoscopies performed (approximately 30%), and proportionate decreased detection of adenoma and cancer 4, 5. There has also been an increased elective surgical waiting time due to lockdown measures 6. 

POCUS has proved to be an accurate technique for the diagnosis of gastrointestinal cancers 7, 8. The diagnosis of colon carcinoma on ultrasonography is made based on the sonographic finding of a localised irregular colon wall thickening or a hypoechoic mass with a hyperechoic centre (Pseudokidney sign 1). The Pseudokidney sign, also called the rosette sign, is demonstrated by segmental hypoechoic bowel wall thickening (due to edema) with a central irregular hyperechoic lumen (due to air and mesentery). The term “Pseudokidney” came about as the lesion appears sonographically as a mass with a reniform appearance. It should be noted that although the pseudokidney sign was first described in colon carcinoma, it has also been described in many other entities such as intussusception as well as in a variety of gastrointestinal diseases. Any gastrointestinal disease (e.g. necrotising enterocolitis, volvulus, lymphoma, inflammatory bowel disease) that leads to hypoechoic circumscribed wall thickening around the hyperechoic pattern of bowel lumen can present with a kidney-shaped appearance by sonography 9. Nonetheless, the detection of a pseudokidney points towards a serious intestinal pathology which accelerates the decision to proceed with endoscopic evaluation.

POCUS is a technique that is non-invasive, cheap, accessible and does not require excessive time to perform. It is also risk free and can be applied repeatedly even for pregnant women and children. In our setting, patients come in without any preparation and tolerate the procedure well. Nonetheless, a negative POCUS exam does not exclude the need for endoscopic examination since rectal tumours are not easily detected on transabdominal ultrasonography. In patients such as ours with non-specific clinical symptoms, POCUS works well as the first diagnostic approach and enables clinicians to prioritise which patients get their endoscopic evaluations earlier. 

## Conflict of interest

The authors have no conflict of interest to declare.

## Funding

The authors received no funding.

## Patient consent

The authors gained consent from the patient to publish.
